# The Cool Factor: Season Modifies Cardiorespiratory Deaths in China

**Published:** 2008-09

**Authors:** Carol Potera

Outdoor air pollution has been linked with increased risk of death from cardiorespiratory disease in epidemiologic studies in North America and Europe. Some studies have found that sex, age, or other modifying factors can cause increased susceptibility to air pollution in some individuals. However, few of these studies have been conducted in Asia. Now a new study of Shanghai residents reveals that the elderly, women, and individuals with lower educational backgrounds are especially vulnerable to outdoor air pollution during cooler weather **[*EHP* 116:1183–1188; Kan et al.]**.

The researchers examined death certificates recorded between 1 January 2001 and 31 December 2004 in the central area of Shanghai and found an average of 119 nonaccidental deaths reported daily, with 49.1% due to cardiorespiratory disease. They collected daily air pollution data for particulate matter less than 10 μm in diameter (PM_10_), sulfur dioxide (SO_2_), nitrogen dioxide (NO_2_), and ozone (O_3_) from the Chinese government agency that tracks air pollutants and assessed how mortality and pollutant levels varied by sex, age, educational status, and season of the year.

They found that most air pollutant levels peaked in the cool season (October through March, when the temperature averages 58°F), correlating with a peak in the death rate; the exception was O_3_, which had higher concentrations in the warm season (April through September, when the temperature averages 75°F). They observed a 2- to 3-times greater risk of death from cardiorespiratory disease in the cool season compared with the warm season, with SO_2_, NO_2_, and O_3_ particularly showing seasonal differences in association with cause of death. The same air pollutants were also associated with a 3- to 4-fold greater risk of cardiovascular death in the cool season than in the warm season, possibly because exposure to air pollutants is reduced by staying inside air-conditioned buildings.

Additionally, people older than 65 were up to 5 times more likely than younger people to die of cardiorespiratory disease. Compared with men, deaths in women were twice as likely to be linked to elevated O_3_ and PM_10_ levels. This may be due to men’s greater rate of smoking, the effects of which may override pollution-related effects in male smokers. Overall, people with less education were twice as likely as more educated residents to die during periods of elevated pollution. Educational level, a reflection of socioeconomic status, has been reported previously as a modifying factor for air pollution–related deaths in North America and Europe, but this is the first such report from mainland China, where the concentrations of PM_10_, SO_2_, and NO_2_ are much higher.

## Figures and Tables

**Figure:**
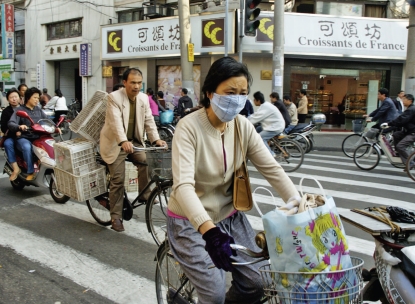
A study of Shanghai residents showed that cardiorespiratory deaths increased during the cool season, which runs from October through March.

